# 1892. Anti-Tubercular Drug-Induced Liver Injury in Patients with Fatty Liver

**DOI:** 10.1093/ofid/ofad500.1720

**Published:** 2023-11-27

**Authors:** Radhika Sarda, Manish Soneja, Shalimar, Surabhi Vyas, Sada Dwivedi, Pankaj Jorwal, Piyush Ranjan, Naval Vikram, Ashutosh Biswas, Naveet Wig

**Affiliations:** All India Institute of Medical Sciences, Delhi, Delhi, India; All India Institute Of Medical Sciences, Delhi, Delhi, India; All India Institute of Medical Sciences, Delhi, Delhi, India; All India Institute of Medical Sciences, Delhi, Delhi, India; All India Institute of Medical Sciences, Delhi, Delhi, India; All India Institute of Medical Sciences, Delhi, Delhi, India; All India Institute of Medical Sciences, New Delhi, New Delhi, Delhi, India; All India Institute of Medical Sciences, Delhi, Delhi, India; All India Institute of Medical Sciences, Delhi, Delhi, India; All India Institute of Medical Sciences, Delhi, Delhi, India

## Abstract

**Background:**

Anti-tubercular drugs are known to cause drug-induced liver injury (DILI) but limited data is available to highlight risk factors for the development of ATT - DILI. Non alcoholic fatty liver disease (NAFLD) has become a leading cause of chronic liver disease. The study aims to bring out incidence and risk factors of ATT-DILI with particular focus on NAFLD.

**Methods:**

This was a prospective cohort study done at a tertiary care centre in North India. Primary objective was to estimate the incidence of ATT-DILI in patients with fatty liver. Other objectives were to compare the incidence of ATT-DILI in patients with and without fatty liver and to study other risk factors for ATT-DILI. All outpatients being initiated on Category I ATT were screened. Fibroscan or Ultrasound abdomen was done to look for the presence of fatty liver. Serial liver function tests were done and patients were followed up till they developed DILI or 2 months whichever was earlier (Figure 1).

**Figure 1:**
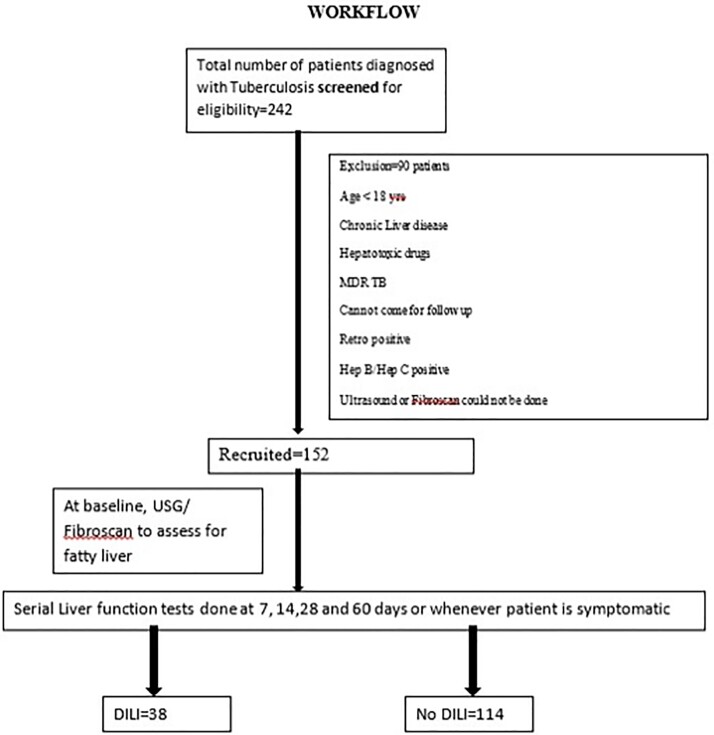
Workflow showing number of patients screened and included in the study

**Results:**

A total of 242 patients were screened and 152 patients were included. The mean age of the study cohort was 35.4 years and 81 patients were male. Sixty out of 152 patients had fatty liver.

The overall incidence of ATT-DILI was 25%. Patients with fatty liver had a significantly higher incidence of DILI as compared to the incidence of DILI in patients who did not have fatty liver (36.6% v/s 17.4%; p-value< 0.001). (Figure 2) Comparing baseline characteristics, patients with DILI had lower serum albumin and higher baseline ALT compared to patients who did not develop DILI. (Table 1) The median time to DILI was 14 days and there was no difference seen in time to DILI in both groups. Presence of fatty liver was associated with development of DILI (Table 2).

Patients who did not have fatty liver had significantly higher incidence of hepatic adaptation as compared to without fatty liver (28.2% v/s 23.3%; p-value=0.04).

**Figure:**
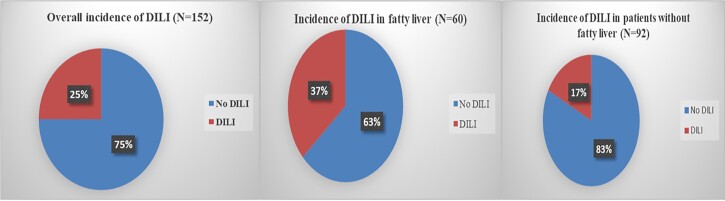

**Figure:**
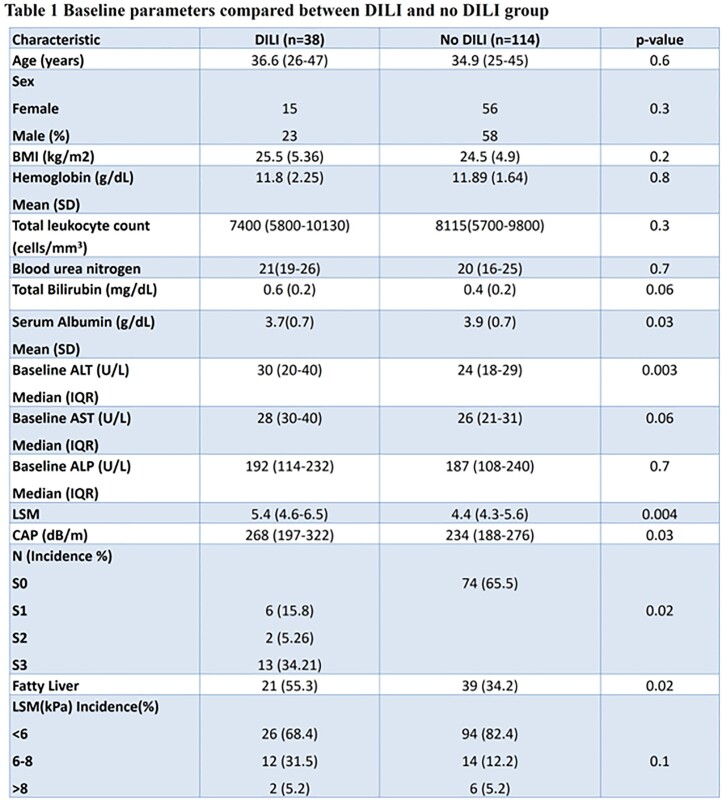

**Figure:**
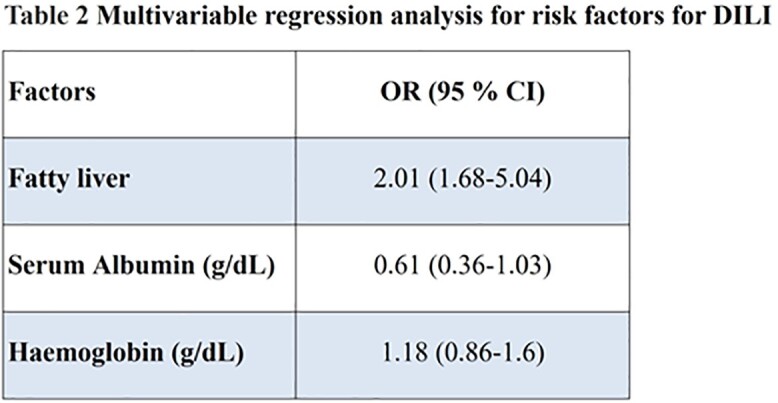

**Conclusion:**

The overall incidence of ATT-DILI is high in our study. Fatty liver was independently associated with development of DILI. Patients with fatty liver need to be monitored closely for development of DILI.

**Disclosures:**

**All Authors**: No reported disclosures

